# Author reply to “Cancer causes emergency home visits”

**DOI:** 10.1002/jgf2.489

**Published:** 2021-08-10

**Authors:** Kaku Kuroda, Taro Miura, Shota Kuroiwa, Moe Kuroda, Naoko Kobayashi, Keiichiro Kita

**Affiliations:** ^1^ Department of family medicine SUNY Upstate Medical University Syracuse NY USA; ^2^ Toyama Machinaka Clinic Toyama Toyama prefecture Japan; ^3^ Department of general medicine University of Toyama Toyama Toyama prefecture Japan

## CONFLICT OF INTEREST

The authors have stated explicitly that there are no conflicts of interest in connection with this article.

To the editor,

We appreciate the author of the letter to the editor^1^ for their critical appraisal of our retrospective observational study, entitled “What are the factors that cause emergency home visits (EHVs) in home medical care in Japan?”.^2^ Kato et al.^3^ reported that the incidence of EHVs in cancer patients was approximately five times higher than that in noncancer patients. They also considered the work of home‐visiting nurses. We agree with them regarding the significance of implementing the duration of the follow‐up period for each case using the person‐time at risk and an offset variable in the Poisson and negative binomial regression to analyze the data considering the weight of the follow‐up period in each case.

We would like to mention two insights.

First, the background of the patient population in their study was different from that in our study. In their study, 23 of 278 patients had cancer. Meanwhile, 124 of 214 patients were in a cancer‐bearing state. Our clinic sees all patients, including stable patients with chronic conditions and complicated cases, both medically and socially. Hence, the severity and performance status of our patients with cancer varied. It is crucial to analyze the rate of EHVs considering the weight of the follow‐up period in each case. Considering the setting of the clinic accepted all patients including end‐stage cancer patients, analyzing the number of EHVs within a certain time period, as in our study, was significant to consider the real‐world incidents and factors of EHVs. Further investigations are needed that include data on the cancer type and severity of patients under their care.

Second, they excluded 166 patients with dementia who did not live with their families, according to the limitation of their study. Our study included all patients in a nursing facility, and a significant number of EHVs were requested from the facility. We agree with them regarding the possibility of underestimating the incidence of EHVs among noncancer patients.

We sincerely appreciated the authors' interest in our article and their constructive comments. Since there has been no direct study on the factors causing EHVs, our study and the study of Kato et al. have contributed to analyzing the incidents and risk factors of EHVs in home medical care in Japan. While there is an urgent need to address both the burden of home‐visiting physicians who are on‐call for 24 h,^4^ and the patients and their families who need to call for an EHV,^5^ there are still insufficient data on these topics. We can collaborate on larger‐scale research and embark on further studies to accumulate data on home medical care.
